# Palatability and Bio-Functionality of Chalky Grains Generated by High-Temperature Ripening and Development of Formulae for Estimating the Degree of Damage Using a Rapid Visco Analyzer of *Japonica* Unpolished Rice

**DOI:** 10.3390/foods11213422

**Published:** 2022-10-28

**Authors:** Sumiko Nakamura, Moeka Hasegawa, Yuta Kobayashi, Chikashi Komata, Junji Katsura, Yasuhiro Maruyama, Ken’ichi Ohtsubo

**Affiliations:** 1 Faculty of Applied Life Sciences, Niigata University of Pharmacy and Applied Life Sciences, 265-1 Higashijima, Akiha-ku, Niigata 956-8603, Japan; 2 NSP Ltd., Nakanoki 2-31-5-B, Funabashi-shi, Chiba 274-0826, Japan

**Keywords:** chalky rice, *α*-amylase activity, proteinase activity, hardness, pasting property

## Abstract

Global warming inhibits grain filling in rice and leads to chalky grains, which are damaged in physical and cooking qualities. In the present paper, we evaluated 54 *Japonica* unpolished rice grains harvested in Japan in 2020, and these samples (original grains) were divided to two groups (whole grains and chalky grains). Using rice grains of 100% whole grains or those blended with 30% of chalky grains, we measured contents of sugars and amino acids, and textural properties of boiled rice grains. It was shown that the α-amylase activity and proteinase activity of raw chalky rice were significantly higher than those of whole rice grains, which led to the significant increase of low-molecular-weight sugars and free amino acids after boiling. Furthermore, hardness and toughness of the boiled rice grains were decreased markedly by blending chalky grains. The ratio of *α*-amylase activity of chalky grains to that of whole grains was shown to be a useful indicator for damage degree by high-temperature ripening. It became possible to estimate the degree of high-temperature damage of rice grains based on only the pasting properties of unpolished rice.

## 1. Introduction

Rice (*Oryza sativa* L.) is one of the most important crops in the world. Global warming is the most serious environmental issue, and high-temperature stress in rice ripening periods causes a decrease in not only grain yield but also quality by generating chalky grains [1]. Nakata et al. [2] showed that the high temperatures accelerating the expression of starch-decomposing α-amylases during ripening is determinative for grain chalkiness. The elucidation of the mechanisms behind grain chalking under high-temperature stress in ripening is indispensable to developing a strategy for preventing the generation of chalky grains in the rice-cultivating region to produce tasty and high-quality rice despite climate warming [3]. In the 1990s, an evasion of high-temperature damage by changing the cultivating period or by using the agronomical method such as deep-water cultivation of rice was attempted. In the 2000s, the mechanisms of high-temperature damage and the location of the related genes to the damage were investigated using a quantitative trait locus (QTL) analysis and a proteome analysis. For example, the metabolism of starch and proteins, oxidative/reductive homeostasis, transcriptive control, mechanisms for prevention, and signal transduction were reported [4,5]. Mitsui et al. [6], Asaoka et al. [7], and Ahmed et al. [8] reported that high temperatures cause the down-regulation of genes for starch synthases and the up-regulation of α-amylases genes, which leads to decreases in the amylose content in endosperm starches of *Japonica* and Basmati rice cultivars. Nakata et al. [2] identified the eight functional α-amylase genes that influence the generation of chalky grains most strongly, which accelerated efficient breeding of high-temperature-tolerant rice cultivars.

As rice is mainly used as a table rice, its quality is very important. Bergman et al. reviewed rice end-use quality analyses, such as the apparent amylose content (AAC) and the rapid visco analyzer (RVA) analysis [9]. The pasting properties of rice are useful indicators in the quality assay of cooked rice and many other processed rice products. Blakeney et al. [10] and Champagne et al. [11] reported that the RVA is useful in evaluating the “degree of cook” after processing of rice into pre-cooked or puffed products. The quality assay of rice is performed by the application of physico-chemical measurements and a sensory test [12].

High-temperature ripening of rice grains causes damage to endosperm starch, which leads to the deterioration in eating properties, such as hardness and stickiness of boiled rice grains and pasting properties of rice flours [13]. In our previous report, we reported the different properties between the whole grains and chalky grains generated in seven *Japonica* unpolished rice grains by high-temperature damage, and we proposed estimation formulae for the retrograded hardness using α-amylase activity and pasting properties as explanatory variables [13]. Nevertheless, simpler estimation formulae for the degree of quality damage by the high-temperature ripening, such as a formula using only RVA, was necessary.

Furthermore, it seems necessary to measure not only change in starch properties but also change in protein and lipid content for the elucidation of the quality change by the high-temperature damage in end-use quality of rice [9]. Similarly, not only the textural properties of the boiled rice but also low-molecular-weight sugars and free amino acids should be measured to elucidate the difference in palatability between whole grains and chalky grains because they are related with the taste of the boiled rice [9,12]. There were few scientific reports about the relationship between high-temperature damage in rice grains and change in proteinase activity, amino acids, fatty acid compositions.

Currently, rice consumers request not only palatable rice but also “healthy rice”, such as brown rice, pigmented rice, and pre-germinated brown rice. For example, fatty acid composition is reported to be important for the bio-functionality of rice.

In this study, we tried to elucidate the quality of rice grains for their proteinase activity, amino acid, and fatty acid effects on high-temperature ripening and to estimate the degree of damage based on the α-amylase activity using an RVA.

To achieve the purpose, we collected and analyzed 54 *Japonica* rice samples with a high ratio of chalky rice grains produced in 2020 when it was extraordinary hot during the summer all over Japan. Furthermore, we tried to develop the estimation formulae for the damage degree of rice grains ripened under high temperatures.

## 2. Materials and Methods

### 2.1. Materials

The unpolished rice samples were purchased in 2020 at a local market and were subjected to the measurement in 2021 (*Japonica* subspecies) (*n* = 54). These original rice samples were divided manually based on the apparent chalkiness to two groups (whole grain and chalky grain): The high-quality premium rice included *Koshihikari* (Niigata A), *Koshihikari* (Niigata B), *Koshihikari* (Shimane), *Koshihikari* (Saga), *Koshihikari* (Fukushima), *Koshihikari* (Yamagata A), *Koshihikari* (Yamagata B), *Koshihikari* (Ibaragi), *Koshihikari* (Toyama), *Koshihikari* (Kyoto), *Koshihikari* (Niigata C), *Koshihikari* (Yamanashi), *Koshihikari* (Niigata D), *Koshihikari* (Niigata E), and *Koshihikari* (Niigata F) (*n* = 15). The ordinary *Japonica* rice included *Shinnosuke* (Niigata), *Hitomebore* (Miyagi), *Oidemai* (Kagawa), *Sasanishiki* (Miyagi), *Kinumusume* (Shimane), *Fufufu* (Toyama), *Akitakomachi* (Akita), *Yumeshizuku* (Saga), *Tsuyahime* (Shimane), *Tsugaruroman* (Aomori), *Sagabiyori* (Saga), *Ginganoshizuku* (Iwate), *Ichihomare* (Fukui), *Morinokumasan* (Kumamoto), *Seitennohekireki* (Aomori), *Toyohashi 1 go* (Aichi), *Sasashigure* (Miyagi), *Hatsushimo* (Gifu), *Tennotsubu* (Fukushima), *Hinohikari* (Saga), *Haenuki* (Yamagata), *Yuudai 21* (Tochigi), *Harumi* (Kanagawa), *Nijinokirameki* (Niigata), *Hitomebore* (Miyagi), *Hoshizoramai* (Tottori), *Tsukiakari* (Niigata), *Kaguyahime* (Miyagi), *Akisakari* (Fukui), *Kazesayaka* (Nagano), *Satojiman* (Kanagawa), *Enmusubi* (Shiga), *Nipponbare* (Shiga), *Nourin 48 go* (Yamanashi), and *Tsuyahime* (Yamagata) (*n* = 35). Low-amylose *Japonica* rice included *Datemasayume* (Miyagi), *Yumepirika* (Hokkaido), *Milky queen* (Yamagata), and *Milky queen* (Kyoto) (*n* = 4). Each sample was stored at 10 °C in rice storage chamber.

### 2.2. Ratios of Whole (Head) and Chalky Rice Grains

We classified the unpolished rice grains into whole grains and chalky ones using an experimental grain inspector (Grain Quality Inspector RGQ120; SATAKE, Corp., Higashihiroshima, Japan) and separated the whole grains and chalky ones visually from 54 rice samples [13].

### 2.3. Preparation of Three Kinds of Brown Rice Flours

Three kinds of unpolished rice flours were from 54 *Japonica* rice samples, which were pre-fractionated original brown rice grains. Concretely, we divided each original rice sample to two groups manually, chalky grains and whole grains, based on the apparent chalkiness. We fractionated chalky grains visually in condition of more than 50% chalkiness based on national inspection standards of agricultural products, Japan. These rice grains were pulverized using a cyclone mill (SFC-S1; UDY, Corp., Fort Collins, CO, USA).

### 2.4. Measurement of the Moisture Content of Rice Flour

The moisture content of the unpolished rice flours was measured using an oven-drying method with slight modification by drying 2 g flour samples for 1 h at 135 °C [14].

### 2.5. Preparation of Starch Granules

Starch granules were purified from whole and chalky unpolished rice flours using cold alkaline solution according to the method reported by Yamamoto et al. [15].

Milled rice flour (2.0 g) was suspended in 30 mL of a 0.1% sodium hydroxide solution and vibrated by a water bath at 0 °C for 3 h at 100 min^−1^ (100 rpm). The suspension was then centrifuged for 5 min at 1500× *g*, the supernatant layer was removed, and the precipitate was suspended by adding 40 mL of distilled water. The solution was then vibrated by a water bath at 0 °C for 0.5 h at 100 vibrations min^−1^. After centrifugation for 5 min at 1500× *g* and removal of the supernatant layer, the process was repeated three times. A check of pH neutrality was then conducted using pH test paper. After the precipitate was re-suspended in 30 mL of 60% ethanol solution, it was vibrated by a water bath at 0 °C for 0.5 h with reciprocating motions (100 turns/min). After centrifugation for 5 min at 1500× *g*, the supernatant layer was removed and the precipitate was suspended by adding 30 mL of an acetone solution and vibrated by a water bath at 0 °C for 0.5 h at 100 vibrations min^−1^. After centrifugation for 5 min at 1500× *g*, the supernatant layer was removed and the precipitate was dried at room temperature.

### 2.6. Pasting Properties

The pasting properties of pre-fractionated original unpolished rice flours, fractionated whole unpolished rice flours, or the fractionated chalky unpolished rice flours from 54 *Japonica* rice samples were measured using a Rapid Visco Analyzer (RVA) (model Super 4; Newport Scientific Pty Ltd., Warriewood, Australia) [16,17].

### 2.7. Iodine Absorption Spectrum

The iodine absorption spectrum of alkali-treated whole and chalky starch flours was measured using a Shimadzu UV-1800 spectrophotometer. The AACs of alkali-treated rice starch were measured using the iodine colorimetric method of Juliano [18,19,20].

### 2.8. α-Amylase Activity

*α*-amylase activity of the whole and chalky unpolished rice flours was determined by the enzyme kit (Megazyme International Ireland, Ltd., Wicklow, Ireland).

### 2.9. Polishing and Boiling of Rice Samples

We prepared polished rice (milling yield of 90–91%) using an experimental friction-type rice milling machine (Yamamotoseisakusyo Co., Yamagata, Japan). Then, 10 g of the polished rice grains of 100% whole rice grains (whole rice) and 30% chalky grains blended rice (blend rice) were boiled according to the method of our previous report [13]. *Koshihikari* (Saga), *Koshihikari* (Ibaragi), *Koshihikari* (Niigata E), *Koshihikari* (Niigata F), *Shinnosuke* (Niigata), *Hitomebore* (Miyagi), *Oidemai* (Kagawa), *Sasanishiki* (Miyagi), *Kinumusume* (Shimane), *Fufufu* (Toyama), *Akitakomachi* (Akita), *Yumeshizuku* (Saga), *Tsuyahime* (Shimane), *Tsugaruroman* (Aomori), *Sagabiyori* (Saga), *Ginganoshizuku* (Iwate), *Ichihomare* (Fukui), *Morinokumasan* (Kumamoto)*,* and *Seitennohekireki* (Aomori) rice were used as rice samples.

### 2.10. Measurements of Textural Properties of Boiled Rice Grains

Textural properties of the samples (10 g) were measured by the bulk measurement using a Tensipresser (My Boy System, Taketomo Electric Co., Tokyo, Japan) according to the method described by Odahara et al. [21]. The bulk measurements were repeated five times, and the mean value was calculated.

### 2.11. Measurement of d-Glucose, Maltose and Saccharose Contents

The cooked rice flour sample was prepared by pulverization after lyophilization.

d-glucose of each sample (0.1 g) was extracted by shaking with 1 mL of 60% ethanol at room temperature for 1 h and measured by the enzyme assay method (F-kit, Roche/R-Biopharm AG., Darmstadt, Germany).

### 2.12. Measurement of l-Glutamic Acid

The cooked rice flour sample was prepared by pulverization after lyophilization.

l-glutamic acid was extracted from each sample (0.1 g) by shaking with 1 mL of 60% ethanol at room temperature for 1 h and then measured by the enzyme assay method (F-kit, Roche/R-Biopharm AG., Darmstadt, Germany).

### 2.13. Measurement of l-Amino Acid

The cooked rice flour sample was prepared by pulverization after lyophilization.

Each sample (0.1 g) was extracted by shaking with 1 mL of 10 mM PBS buffer (pH 7.0) at 4 °C for 1 h and then centrifuged for 15 min at 3000× *g*. After that, the supernatant of the extraction solution (50 μL) was measured by (50 μL) of reaction mix buffer (0.5 μL fluorometric probe, 0.1 μL horseradish peroxidase catalyzes, 1.7 μL l-Amino acid Oxidase, and 47.7 μL × 1 Assay B) at 37 °C for 2 h using l-Amino Assay kit (Fluorometric, CELL BIOLABS, INC., San Diego, CA, USA). The sample l-Amino Acid concentrations were determined by comparison with a known L-Alanine standard.

### 2.14. Analysis of Rice Protein Composition (SDS-PAGE)

The proteins were analyzed using SDS-PAGE described in the report using 12% polyacrylamide gel [13]. We used ATTO densitograph software library (CS Analyzer ver. 3.0, ATTO CORPORATION, Toyko, Japan) to calculate the intensities of various spots on the gel after SDS-PAGE.

### 2.15. Protease Activity

Protease activities of the whole and chalky unpolished rice flours were determined by the Amplite^TM^ Universal fluorometric protease activity assay kit Green Fluorescence (AAT Bioquest, Inc., Sunnyvale, CA, USA). For activity measurements, proteases were extracted from *Koshihikari* brown rice flour (0.5 g) with 2 mL of extraction buffer (20 mM Tris-HCl, pH 6.8, 50 mM NaCl, 5 mM CaCl_2_) at 5 °C for 16 h. After centrifugation for 15 min at 3000× *g,* the supernatant of the extraction solution was subjected to lyophilization. These freeze-dried samples (0.009 g) were dissolved in 180 μL of de-ionized water, and those solutions (50 μL) and substrates (casein labeled with a fluorescent dye) (50 μL) were mixed and incubated at 37 °C for 50 min in a 96-well solid black microplate, and protease activity was determined by the fluorometric method (Grating Based Multimode Reader SH-9000: Corona Electric Co, Ltd., Hitachinaka, Japan). The experiments were repeated three times.

### 2.16. Statistical Analyses

We used Excel Statics (ver. 2006; Microsoft Corp., Tokyo, Japan) for the statistical analysis of the significance of regression coefficients using Student’s *t*-test, one-way analysis of variance, and Tukey’s test. Additionally, the method of Tukey’s multiple comparison was statistically analyzed using Excel NAG Statistics add in 2.0 (The Numerical Algorithms Group Ltd., Tokyo, Japan).

## 3. Results and Discussion

### 3.1. Ratio of Whole Rice Grains in 54 Japonica Rice Samples in 2020

According to the report of the Japan Meteorological Agency, there were many extremely hot days with a temperature of more than 35 °C in 2020. The ratios of the whole unpolished rice grains in low-amylose *Japonica* rice (12.1–49.9%; mean, 30.4%) were lower than those in 35 ordinary *Japonica* rice (5.6–83.2%; mean, 58.0%) and those in 15 premium rice, *Koshihikari* (44.3–65.0%; mean, 56.0%) (data not shown). Those of rice samples were damaged by a minimum temperature of higher than 25°C from evening to next morning in August 2020.

### 3.2. Pasting Properties

Rapid Visco Analyzer (RVA) is commonly used for the evaluation of physicochemical properties of the starches as pasting characteristics [12,22]. Starch is essentially composed of amylose and amylopectin. Starch is composed of amylose and amylopectin. The former is small and linear molecule, whereas the latter is a large and highly branched one in the form of amylose–lipid complexes (ALCs) [23,24]. In our previous paper, we found that it is possible to estimate the fatty acid composition based upon the pasting properties measured by an RVA [25].

Pasting properties are useful quality indicators because they affect the eating quality of rice [9,12,22]. The final viscosity (Fin. vis) and consistency (Cons) are related to the degree of starch retrogradation during cooling [24]. In the previous study, we found a novel index of the ratios of setback/consistency (SB/Cons) and Max. vis/Fin. vis (Max/Fin), which positively or negatively correlated with the proportion of intermediate- and long-chains of amylopectin (Fb_1+2+3_ (DP ≥ 13)) [17].

Table 1 and Supplemental Tables S1–S3 show that the Fin. vis and Cons of low-amylose *Japonica* rice were significantly lower than premium and ordinary *Japonica* rice.

**Figure 1:**
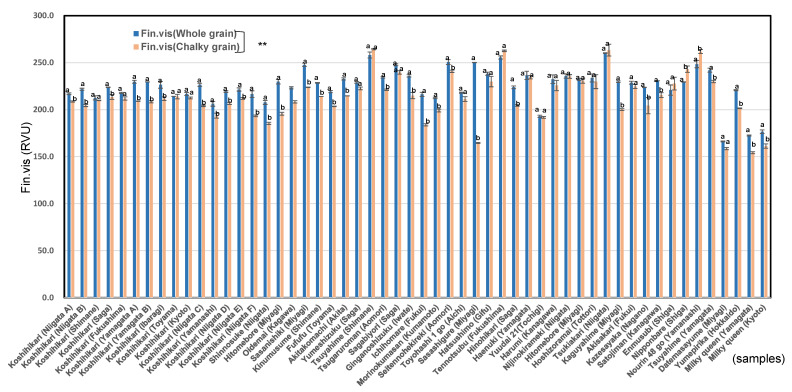
Final.viscosity of whole and chalky rice grains in 54 *Japonica* unpolished rice samples in 2020. Different letters (a, b) mean that whole and chalky grains in each same rice samples are significantly different. ** Correlation is significant at 1% by the method of Tukey’s multiple comparison.

Supplemental Tables S1–S3 shows that the Max. vis (maximum viscosity) of chalky rice grains of premium *Japonica* rice, ordinary *Japonica* rice, and low-amylose *Japonica* rice were significantly lower than those of whole rice grains. The pasting properties of ordinary *Japonica* rice showed a similar tendency as whole rice grains, whereas those of original rice grains of premium *Japonica* rice showed a little higher than that of whole rice grains, while those of low-amylose *Japonica* rice of original rice showed a little lower than that of whole rice grains. As shown in Figure 1, almost all chalky rice grains were significantly lower than those of whole rice grains at Fin. vis (*p* < 0.01 **) and Cons (*p* < 0.05 *).

In the previous study, we developed a novel estimation formula for linoleic acid, oleic acid contents, and a ratio of omega-6 fatty acids to omega-3 fatty acids (n-6/n-3) based upon the pasting properties of *Japonica* brown rice cultivars [25,26].

**Figure 2:**
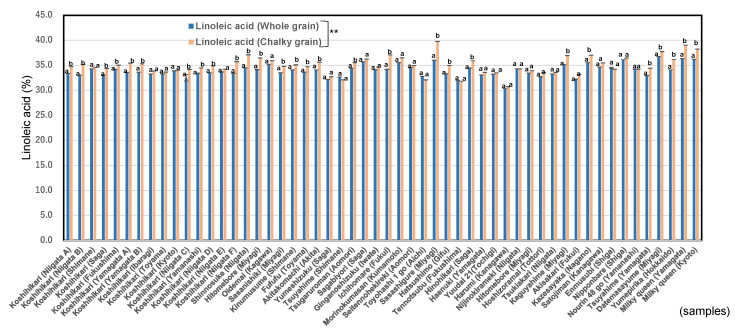
Comparison in linoleic acid between whole and chalky rice grains in 54 *Japonica* rice in 2020. Different letters (a, b) mean that whole and chalky grains in each same rice samples are significantly different. ** Correlation is significant at 1% by the method of Tukey’s multiple comparison. Formula for estimating the fatty acid content based on the pasting properties of brown rice [25].

Figure 2 shows that the whole rice grains contained less linoleic acid than the chalky rice grains significantly (*p* < 0.01), and Supplemental Figure S1 shows n-6/n-3 values of chalky rice grains were significantly higher than those of whole rice grains (*p* < 0.01). ANOVA showed significant difference (*p* < 0.01) by comparing all 54 chalky grains and whole grains.

Simopoulos et al. [27] showed that a low n-6/n-3 ratio exert suppressive effects to pathogenesis of several diseases, whereas Western diets showed an excessive amount of ones. According to this report [27] and our results, it seems that the fatty acid composition in brown *Japonica* rice were deteriorated by high temperatures during ripening.

Supplemental Figure S1 shows that the high-temperature ripening affects not only eating/processing qualities but also the bio-functionality through the change in fatty acid compositions.

Taira et al. [28] reported that lipid content and fatty acid composition of rice were affected by the temperature during ripening. The dissociation temperature of ALCs increased with an increase in chain length of the fatty acids. Nevertheless, dissociation enthalpy was practically independent of chain length [29,30].

As a result, it seems that pasting properties and fatty acid of almost all *Japonica* rice samples were affected by high temperatures during ripening, and degree of damage shows varietal differences. It is estimated that low-amylose *japonica* rice is more susceptible to high-temperature damage than premium and ordinary *japonica* rice cultivars.

Linoleic acid content = −0.044 × Max. vis − 10.1 × Set/Con + 0.108 × SB + 47.602.(1)

### 3.3. Iodine Absorption Spectrum

It has been reported that most genes are markedly influenced by high temperature during the ripening of rice grains, either up-regulated or down-regulated [31].

It was reported that the high-temperature-ripened grains contained decreased levels of amylose and long chain-enriched amylopectin, which might arose from the repressed expression of granule bound starch synthase (GBSS) and branching enzymeIIb (BEIIb), respectively [32]. Low-amylose rice generally becomes soft and sticky after cooking, whereas high-amylose rice becomes hard with fluffy separated grains [33,34]. The starches in the rice grains grown under low temperature have higher amylose content and lower SLC (super-long chains) amylopectin content [35]. Inouch et al. [36] showed that the SLC content of starch can be estimated on the basis of λ_max_ and the blue value of purified amylopectin. Furthermore, Igarashi et al. [37] reported a positive correlation between absorbance at λ_max_ and apparent amylose content (AAC).

Table 2 and Supplemental Tables S4 and S5 show that the absorbance values around 620 nm (AAC) of the chalky rice grains of low-amylose *Japonica* rice were lower than those of whole rice grains. As a result, whole rice grains showed higher AAC values than chalky grains significantly (*p* < 0.05). Moreover, λ_max_ and Aλ_max_ values of chalky rice grains in low-amylose rice were lower than those of whole rice grains similarly with AAC. It seems that starch synthase activities were lower and amylase activities were higher in the chalky grains than those in the whole grains in low-amylose rice group.

In the previous study, we developed the λ_max_/AAC ratios as novel index for the degree of damage by high temperature [13]. The λ_max_/A_λmax_ ratios of the chalky rice grains of premium *Japonica* rice were significantly higher than those of whole rice grains.

It seems that AACs of low-amylose rice samples were affected and lowered by high temperature during ripening. The ratios of AACs of whole grains to those of chalky rice grains in the case of low-amylose *Japonica* rice (1.13 ± 0.0) were higher than those of premium *Japonica* rice (1.07 ± 0.1) and ordinary *Japonica* rice (1.05 ± 0.1).

It was reported that the high-temperature damage for low-amylose rice is accelerated by alleles located at dull 1~5, and those of five dull loci, which leads to lowering the amylose content [38,39,40]; moreover, these low-amylose rice cultivars have both genes of Wx, which causes stronger effects by high temperature during the ripening period than ordinary non-glutinous rice cultivars [32].

### 3.4. α-Amylase Activity

**Figure 3:**
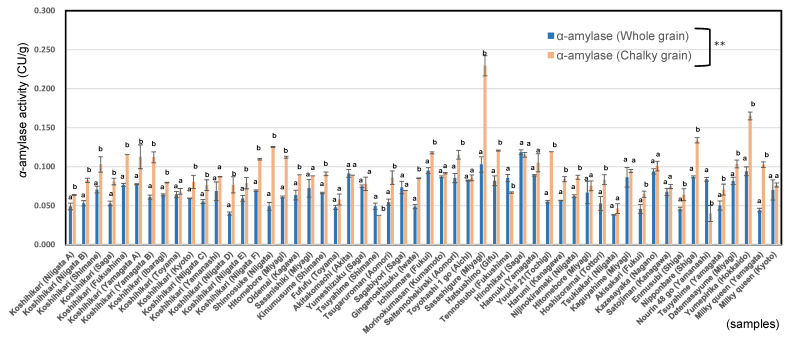
α- amylase activities of whole and chalky unpolished rice grains in 54 *Japonica* rice in 2020. Different letters (a, b) mean that whole and chalky grains in each same rice samples are significantly different. ** Correlation is significant at 1% by *t*-test by the method of Tukey’s multiple comparison.

Nakata et al. [2] showed that the promoter activity of most α-amylase genes was elevated at high temperature. Mitsui [3] and Yamakawa et al. [41,42] reported that α-amylase is a key factor in grain chalkiness using transgenic studies of ectopic overexpression and suppression of α-amylase. As shown in Figure 3, the α-amylase activities of chalky unpolished rice grains of premium *Japonica* rice Koshihikari, ordinary *Japonica* rice, and low-amylose *Japonica* rice were significantly higher than those of whole rice grains. As a result, whole unpolished rice grains were shown to have significantly lower α-amylase activities than chalky unpolished rice grains.

The ratios of α-amylase activities of chalky unpolished rice grains to whole unpolished rice grains in low-amylose rice (1.61 ± 0.1) was higher than those of premium rice Koshihikari (1.41 ± 0.2) and ordinary *Japonica* rice cultivars (1.32 ± 0.3). Chalky unpolished rice grains showed markedly higher α-amylase activities (1.3–1.7 times) than whole unpolished rice grains in 54 *Japonica* rice in 2020 (data not shown).

Our results show that α-amylase activity of chalky unpolished rice grains are higher than those of whole unpolished rice grains in accordance with the reports by other researchers [3,42,43]. Additionally, it was found that the tendency is stronger in the low-amylose rice group.

### 3.5. Correlation between Pasting Properties of Original Rice and Ratios of α-Amylase Activity of Chalky Grains to Those of Whole Rice Grains

The global warming rates of 1.5 °C and 2 °C may be exceeded during the 21st century [44].

In a previous study, we found an novel index for RS content, the ratios of Max/Mini and Max/Fin, which have stronger negative correlations than the conventional indexes reported using an RVA [16].

Hakata et al. [45] proposed that the suppression of α-amylase genes is a potential strategy for ameliorating grain damage from global warming. One of the reasons why α-amylase is very important may be that α-glucosidase is predominantly localized in the inner endosperm [46], whereas α-amylase is localized mainly in the outer layers.

The α-amylase activities of chalky unpolished rice grains were much higher than those of whole unpolished rice grains in 54 *Japonica* rice in 2020. Particularly, low-amylose rice and premium rice Koshihikari in Niigata showed very high values.

The whole and chalky grains have the same genes, and those of ratios of α-amylase activites of chalky unpolished grains to those of whole ones were shown as an index of degree of damage by high-temperature ripening [6] because the chalky unpolished rice grains of almost all *Japonica* rice were significantly higher than those of whole unpolished grains.

Table 3 shows that the ratios of α-amylase activities of chalky unpolished grains to those of whole ones of 50 *japonica* rice except low-amylose rice showed negative correlations with Mini. vis (r = −0.51; *p* < 0.01), Fin. vis (r = −0.60; *p* < 0.01), and Cons (r = −0.45; *p* < 0.01) and a positive correlation with Max/Mini (r = 0.35; *p* < 0.05) of pasting properties of (before dividing to two groups) original 50 *Japonica* rice.

To summarize, the ratios of α-amylase activites of chalky unpolished grains to those of whole ones of almost all *Japonica* rice except low-amylose rice showed a high correlation with pasting properties of original *Japonica* rice.

### 3.6. Formula for Estimating the Ratios of α-Amylase Activities of Chalky Unpolished Grains to Those of Whole Ones Based on Only the Pasting Properties in Original *Japonica* Rice Using an RVA

In our previous paper, we reported that the pasting properties, measured by the program at 120 °C using an RVA, were useful to estimate the retrogradation degree of hardness of the boiled rice grains [47]. In the present paper, we developed a novel estimation formula for the degree of high-temperature damage based on only the pasting properties by an RVA.

**Figure 4:**
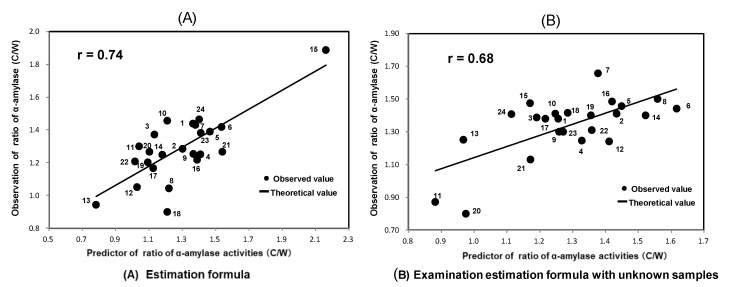
The estimating formula for the ratios of α-amylase activities of chalky unpolished grains to those of whole ones of *Japonica* rice except low-amylose rice based on the pasting properties in original *Japonica* rice using RVA. (**A**) Estimation formula; (**B**) Examination estimation formula with unknown samples. C, chalky grains; W, whole grains; ratios of α-amylase activities (C/W), ratios of α-amylase activities of chalky grains to those of whole ones.

Figure 4A shows the formula for estimating the ratios of α-amylase activities of chalky unpolished grains to those of whole ones of 24 original Japonica brown rice based on the pasting properties of original *Japonica* brown rice using an RVA.

The equation had a correlation coefficient (r) of 0.74 in the calibration. The following formula for estimating the ratios of α-amylase activities of chalky grains to those of wholes of 24 original brown *Japonica* rice.

Ratios of α-amylase activities (C/W) = −0.01 × Fin. vis + 0.33 × Max/Mini + 2.69.(2)

where C, chalky grains; W, whole grains; and ratios of α-amylase activities (C/W), ratios of α-amylase activities of chalky grains to those of whole ones.

Figure 4B shows that a correlation coefficient (r) of 0.68 was obtained with the application of the abovementioned formula for the validation test using 24 unknown samples.

Thus, the validation test showed that the equation can be applied to unknown samples. In the whole and chalky unpolished rice grains with the same genotype, the chalky unpolished rice grains of almost all *Japonica* rice have significantly higher α-amylase activities than the whole unpolished grains on high-temperature ripening. Therefore, α-amylase activities could be a good index for the degree of high-temperature damage. As a result, it became possible for us to estimate the degree of damage by high-temperature ripening, using only the pasting properties of original (mixture of whole and chalky grain) *Japonica* unpolished rice except low-amylose samples because the enhancement of α-amylase activities had been reported to be a good index for high-temperature damage [6,13].

(A) Estimation formula: Estimation formula: The following formula for estimating the ratios of α-amylase activities of chalky unpolished grains to those of whole ones of 24 original *Japonica* rice except low-amylose rice. 1, Koshihikari (Niigata A); 2, Koshihikari (Saga); 3, Koshihikari (Fukushima); 4, Koshihikari (Yamagata); 5, Koshihikari (Kyoto); 6, Koshihikari (Niigata D); 7, Koshihikari (Niigata F); 8, Sasanishiki (Miyagi); 9, Kinumusume (Shimane); 10, Fufufu (Toyama); 11, Yumeshizuku (Saga); 12, Sagabiyori (Saga); 13, Tennotsubu (Fukushima); 14, Haenuki (Yamagata); 15, Yuudai 21 (Tochigi); 16, Nijinokirameki (Niigata); 17, Hitomebore (Miyagi); 18, Tsukiakari (Niigata); 19, Kaguyahime (Miyagi); 20, Satojiman (Kanagawa); 21,
(B) Examination estimation formula with unknow samples: The formula for validation test using 24 unknown samples.1, Koshihikari (Niigata B); 2, Koshihikari (Shimane); 3, Koshihikari (Yamagata); 4, Koshihikari (Ibaragi); 5, Koshihikari (Toyama); 6, Koshihikari (Yamanashi); 7, Koshihikari (Niigata E); 8, Shinnosuke (Niigata); 9, Hitomebore (Miyagi); 10, Oidemai (Kagawa); 11, Tsuyahime (Simane); 12, Ichihomare (Fukui); 13, Seitennohekireki (Aomori); 14, Toyohashi 1 go (Aichi); 15, Hatsushimo (Gifu); 16, Harumi (Kanagawa); 17, Hoshizoramai (Tottori); 18, Akisakari (Fukui); 19, Kazesayaka (Nagano); 20, Nourin 48 go (Yamanashi); 21, Ginganoshizuku (Iwate); 22, Morinokumasan (Kumamoto); 23, Koshihikari (Niigata C); 24, Tsuyahime (Yamagata). The formula was developed to estimate the degree of damage by high-temperature ripening based on the pasting properties of original *Japonica* unpolished rice except low-amylose rice using RVA, of which the correlation coefficient was 0.74 for calibration and 0.68 for the validation test.

### 3.7. Textural Properties of Boiled Rice Grains

In our previous report, boiled rice grains from the chalky grains showed lower hardness and higher retrogradation degree after boiling compared with the whole grains [13,19].

In the recent commercial market, rice grains containing about 30% of chalky rice are graded as low class and have prices lower than whole rice grains. Markedly, damaged rice samples in our 54 rice samples contained about 30% of chalky rice.

In this study, we measured the physical properties of the boiled rice of whole rice (100% whole grains) and blend rice (30% chalky grains blended ones) of 19 *Japonica* rice by the bulk measurement (10 g) with a Tensipresser. Although we used single grain method in our previous papers, we adopted “bulk method” in order to clarify the effect of blending the chalky rice grains. The value of Hardness is indicated by the height and that of Toughness is area for continuous progressive compression in Tensipresser [48].

**Figure 5:**
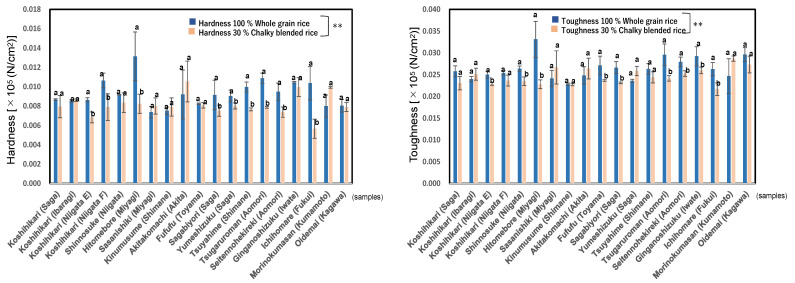
Physical properties of boiled rice of 100% whole rice grains and 30% chalky rice blended grains in 19 kinds of *Japonica* rice in 2020. Different letters (a, b) mean that whole and 30% chalky grains in each same rice samples are significantly different. ** Correlation is significant at 1% by the method of Tukey’s multiple comparison.

As shown in Figure 5, the hardness of blended boiled rice (0.0057–0.0106 × 10^5^ N/cm^2^; mean, 0.0081) was significantly lower than that of whole boiled rice (0.0074–0.0131 × 10^5^ N/cm^2^; mean, 0.0093 × 10^5^ N/cm^2^), at *p* < 0.01, and toughness showed a similar tendency, at *p* < 0.01. The hardness and toughness of blended boiled rice were lower (0.87~0.93 times) than those of whole boiled rice grains.

As shown in Supplemental Tables S6 and S7, the stickiness of blended boiled rice (0.0242–0.0364 × 10^5^ N/cm^2^; mean, 0.0282) were a little lower than those of whole boiled rice (0.0239–0.0341 × 10^5^ N/cm^2^; mean, 0.0300 × 10^5^ N/cm^2^), and those of adhesion showed a similar tendency. The stickiness and adhesion of blended boiled rice were lower (0.94~0.98 times) than those of whole boiled rice grains.

We ascertained that blended rice grains showed a little lower hardness and stickiness than whole rice grains after boiling, which means the physical properties of blended boiled rice are inferior to whole rice grains in terms of eating quality. This means that the practical or commercial rice grains (about 30% chalky rice blend) in the market may be inferior to the un-damaged rice grains in terms of textural quality.

As shown in Supplemental Table S8, the stickiness of blended boiled rice showed a positive correlation with α-amylase activity of chalky grains (r = 0.81, *p* < 0.01). Moreover, the hardness of blended boiled rice showed a positive correlation with the total oligo saccharides (r = 0.50, *p* < 0.05), saccharose (r = 0.58, *p* < 0.05), and maltose (r = 0.56, *p* < 0.05) of the blended rice. It means that the acceleration of amylase affects rice quality markedly in the case of high-temperature ripening.

### 3.8. d-Glucose, Maltose, and Saccharose Contents in Boiled Rice Grains

Awazuhara et al. [49] showed that the thermal dependency and stability of enzymes producing reducing sugar were different between outer endosperm and inner endosperm of rice. The amounts of reducing sugars were involved by multiple amylase actions, and those ones showed largest increases at 40–60 °C in during boiling [50].

In this study, we measured the sugar contents of the boiled rice of whole grain rice and blended grain rice of 19 *Japonica* rice samples by UV absorption measurement using the enzymatic method.

As shown in Supplemental Table S9, the d-glucose contents of the blended boiled rice (0.041–0.083%; mean = 0.066%) were about same with those of whole boiled rice (0.041–0.083%; mean = 0.061%). Similarly, the maltose contents of the blended boiled rice (0.085–0.136%; mean = 0.101%) were a little higher than those of whole boiled rice grains (0.075–0.129%; mean = 0.095%).

The saccharose contents of the blended boiled rice (0.282–0.450%; mean = 0.335%) were significantly higher than those of whole boiled rice (0.246–0.396%; mean = 0.299%) at *p* < 0.05.

As shown in Supplemental Table S8, the saccharose contents of whole grains boiled rice showed a positive correlation with the hardness of whole grains ones (r = 0.57, *p* < 0.05).

**Figure 6:**
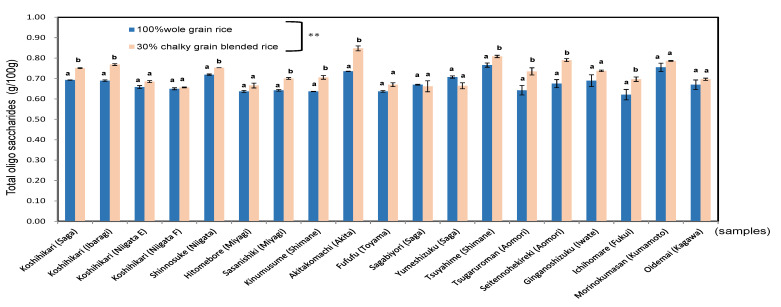
Total oligo saccharides of 100% whole rice boiled grains and 30% chalky rice blended grains in 19 kinds of *Japonica* rice in 2020. Different letters (a, b) mean that whole and 30% chalky grains in each same rice samples are significantly different. ** Correlation is significant at 1% by the method of Tukey’s multiple comparison.

The total oligo saccharides of blended boiled rice (0.662–0.848%; mean = 0.725%) were significantly higher than those of whole boiled rice (0.621–0.755%; mean = 0.678%), at *p* < 0.01 as shown in Figure 6. As a result, the sugar contents of the boiled rice of blended rice were 1.1 times higher than those of whole grains rice. The reason blended boiled rice contains more total oligo saccharides than whole grains could be due to the higher activities of multiple amylases and lower activities of starch synthesizing enzymes [13]. It was confirmed that the sweetness component of boiled rice was increased by blended of 30% chalky grains.

As shown in Supplemental Table S8, the total oligo saccharides of whole grains boiled rice showed a positive correlation with the hardness (r = 0.56, *p* < 0.05) and toughness (r = 0.52, *p* < 0.05) of whole grains boiled ones.

### 3.9. Difference in l-Glutamic Acid

The amino group metastasizes to α-ketoglutaric acid; after that, α-keto acid is produced. Finally, those of all amino groups were collected to glutamic acid. Moreover, the glutamic acid is one of the umami (delicious taste) components.

Generally, the protease activities of germinated cereal seeds are activated. Abe et al. [51] has found an endo-type proteolytic enzyme of the cysteine proteinase class from germinating rice seeds. Doi et al. [52,53] showed that germinating rice contained three carboxypeptidases or carboxypeptidase-like enzymes. Moreover, Tashiro et al. [54] showed that the seeds of corn, foxtail millet, barnyard millet, wheat, barley, and bran of rice have proteinase inhibitor activities.

**Figure 7:**
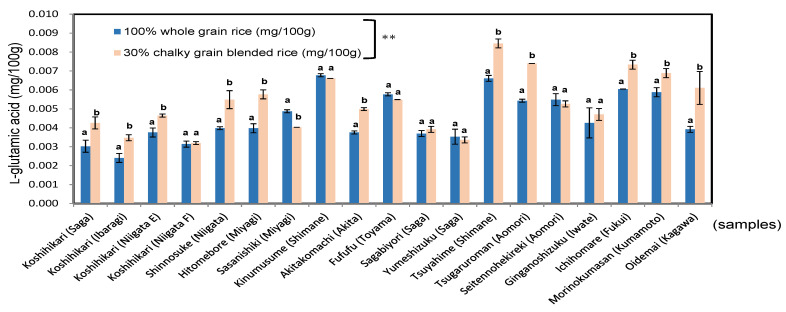
l-glutamic acid of 100% whole rice boiled grains and 30% chalky rice blended grains in 19 kinds of *Japonica* rice in 2020. Different letters (a, b) mean that whole and 30% chalky grains in each same rice samples are significantly different. ** Correlation is significant at 1% by the method of Tukey’s multiple comparison.

As shown in Figure 7, the *l*-glutamic acid content of the blended boiled rice (0.0034–0.0085 mg/100 g; mean = 0.0053 mg/100 g) was significantly higher (1.2 times) than that of whole boiled rice (0.0024–0.0068 mg/100 g; mean = 0.0045 mg/100 g), at *p* < 0.01.

It was presumed that the protease activity of chalky grains is higher than whole grains. It was confirmed that the Umami component of boiled rice was increased by blending 30% chalky grains.

### 3.10. Difference in l-Amino Acids

Tamura et al. [55] showed that the amino acid contents of aspartic acid and glutamic acid are more abundant in the outer than in the inner layers, and those amino acids increased in the cooking water during soaking and increased in the rice grains in the temperature range of 80–100 °C during cooking [50]. Matsuzaki et al. [56] reported the correlation of the glutamic acid and aspartic acid contents with the eating quality of boiled rice, and those of a low level of free amino acid showed a similar tendency in the *Japonica* and *Indica* rice cultivars.

**Figure 8:**
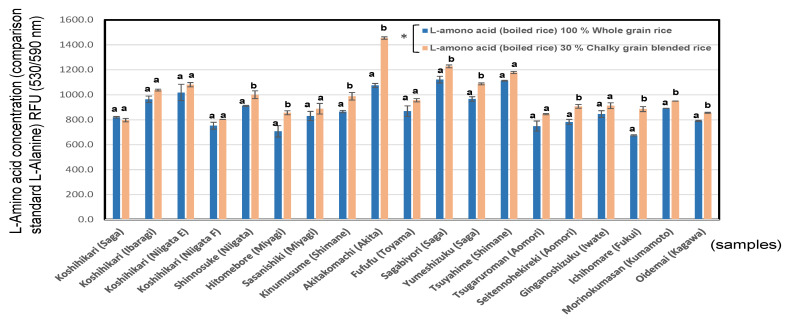
l-amino acid of 100% whole rice boiled grains and 30% chalky blended grains in 19 kinds of *Japonica* rice in 2020. Different letters (a, b) mean that whole and 30% chalky grains in each same rice samples are significantly different. * Correlation is significant at 5% by the Tukey’s multiple comparison method.

As shown in Figure 8, the *l*-amino acid content in the boiled rice of the blended rice (797.2–1455.1 RFU (530/590 nm); mean = 984.9 RFU (530/590 nm)) was significantly higher than that of whole boiled rice (707.7–1121.1 RFU (530/590 nm); mean = 881.4 RFU (530/590 nm)), at *p* < 0.05.

As shown in Supplemental Table S8, the *l*-amino acids in the boiled rice of whole grains showed a positive correlation with the total oligo saccharides (r = 0.62, *p* < 0.01) of whole grains boiled ones.

In the present research, we found that not only starch-related enzymes and sugars but also amino acid contents change markedly in the case of chalky rice grains.

### 3.11. SDS-PAGE of Rice Proteins

The weather conditions influence the protein content in rice grains [57]. It was reported that prolamin contents showed a positive correlation with the hardness of boiled rice grains [2,3,57]. We reported that the 13 kDa prolamin ratios of chalky rice grains were lower than those of whole rice grains [13], and Yamakawa et al. [58] reported a similar tendency.

**Figure 9:**
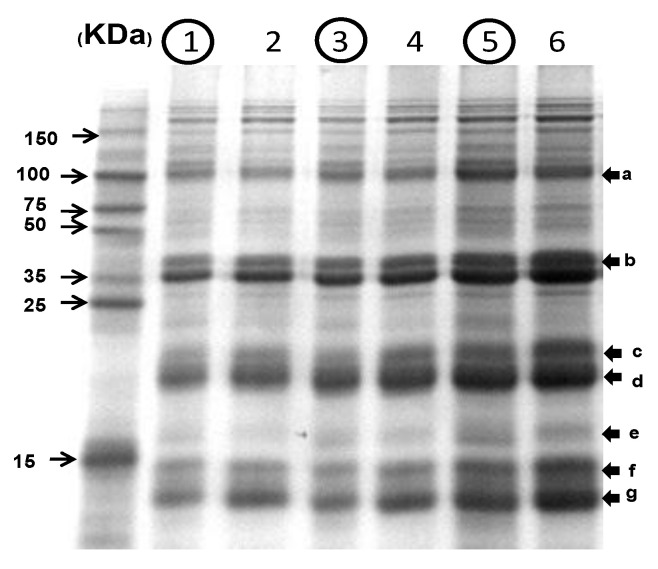
SDS-PAGE analysis of residual proteins extracted from hydrolyzed whole and chalky brown rice flour. 1, hydrolyzed chalky brown rice (Koshihikari) at 37 °C for 16 h; 2, hydrolyzed whole brown rice (Koshihikari) at 37 °C for 16 h; 3, hydrolyzed chalky brown rice (Koshihikari) at 37 °C for 6 h; 4, hydrolyzed whole brown rice (Koshihikari) at 37 °C for 6 h; 5, hydrolyzed chalky brown rice (Koshihikari) at 37 °C for 1 h; and 6, hydrolyzed whole brown rice (Koshihikari) at 37 °C for 1 h. Chalky brown rice grains are expressed in circled numbers; a, gluterin precursor; b, glutelin α-subunit; c, α-globulin; d, glutelin β-subunit; e–g, prolamin.

As shown in Figure 9, the ratios of chalky grains to whole grains in terms of the intensities of the total residual protein bands were 2.00 ± 0.08 after 16 h, 1.65 ± 0.02 after 6 h, and 1.21 ± 0.07 after 1 h for soaking in a buffer solution. As a result, it seems that the protease activity of chalky grains is higher than whole ones. Although many researchers reported that α-amylase activity increases markedly under the high-temperature ripening of rice, there are few reports on the increase in protease activities in chalky rice grains generated under high-temperature ripening. As l-amino acids increase and residual proteins after soaking decrease in the chalky rice grains, we think that high-temperature ripening affects not only starch-related enzymes but also protein-related enzymes in rice grains.

### 3.12. Protease Activity

**Figure 10:**
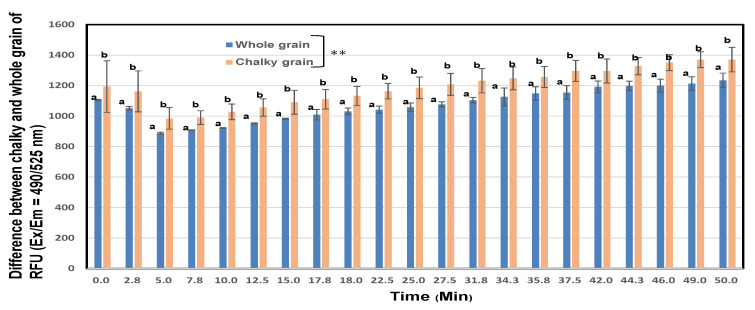
Proteinase activities of unpolished rice flours prepared from whole or chalky unpolished rice grains premium rice Koshihikari. Different letters (a, b) mean that whole and chalky grains in each same rice samples are significantly different. ** Correlation is significant at 1% by the method of Tukey’s multiple comparison.

From the above results in Section 3.11, we measured the protease activities of the whole and chalky unpolished rice by the universal fluorimetricp assay kit. As shown in Figure 10**,** the protease activities of chalky unpolished grains of rice flour (mean = 1193.0 RFU) were significantly higher (*p* < 0.01) than those of whole unpolished grains (mean = 1075.8 RFU). It was presumed that the neutral protease activities of chalky unpolished grains is higher than whole ones because we used buffer on neutral pH, although both of whole and chalky unpolished grains did not show protease activities in the acidic buffer (pH = 3.0).

As we described in Section 3.11, there are few reports on the protease activation under the high-temperature ripening. As we ascertained the increase in protease activities in the chalky unpolished grains, it seems very interesting that not only starch-related enzymes but also protein-related enzymes are activated under the high-temperature ripening of rice grains.

It was reported that protease activities in cereals are enhanced during the germination period similarly to α-amylase activity [59]. Our results reveal that not only α-amylase but also protease are activated by high-temperature ripening. It was reported that the gene expression of gibberellin is closely related with the activation of α-amylase and protease activities [60,61]. Our results are in accordance with these reports at the points of activations of these hydrolytic enzymes by high temperature during ripening.

## 4. Conclusions

Global warming impairs grain filling in rice and leads to chalky-appearing grains, which were damaged in their physicochemical and cooking qualities. In the present paper, we evaluated 54 *Japonica* brown rice grains harvested in Japan in 2020 when it was extraordinary hot during the summer all over Japan from meteorological observation of Japan Meteorological Agency, and these samples (original grains) were divided, manually based on the apparent chalkiness, into two groups (whole grains and chalky grains). The chalky rice grains showed lower values of Max. vis., Mini. vis., BD, Fin. vis, and Cons of pasting properties than the whole rice grains, and their AAC showed a similar tendency, while those of α-amylase activities, protease activities, linoleic acid, oligo saccharide, amino acids, and n-6/n-3 ratio of polyunsaturated fatty acid showed higher than those of whole rice grains. Additionally, we developed a novel estimation formula for the damage degree of rice grains ripened under high temperatures using an RVA.

## Figures and Tables

**Table 1:** Comparison between the pasting properties of chalky, whole, and original rice in 54 *Japonica* unpolished rice in 2020.

	Max. vis	Mini. vis	BD	Fin. vis	SB	Pt	Cons	Set/Cons	Max/Min	Max/Fin
	(RVU)	(RVU)	(RVU)	(RVU)	(RVU)	(°C)	(RVU)			
Premium *japonica* rice (W)	302.5 ± 7.5a	116.8 ± 4.3a	185.7 ± 8.5a	219.9 ± 6.6a	−82.5 ± 9.9a	69.8 ± 0.4a	103.1 ± 2.9a	−0.80 ± 0.11a	2.59 ± 0.11a	1.38 ± 0.05a
Premium *japonica* rice (C)	287.0 ± 11.2b	110.1 ± 3.9a	177.0 ± 11.4b	207.6 ± 6.4b	−79.5 ± 12.4a	70.4 ± 0.7a	97.5 ± 3.5b	−0.82 ± 0.14a	2.61 ± 0.14a	1.38 ± 0.07a
Premium *japonica* rice (O)	313.6 ± 9.5a	121.2 ± 5.7a	192.4 ± 8.2a	229.0 ±8.1a	−84.6 ± 9.4a	69.3 ± 1.3a	107.8 ±3.1a	−0.79 ± 0.10a	2.59 ± 0.11a	1.37 ± 0.05a
Ordinary *japonica* rice (W)	292.8 ± 25.7a	121.9 ± 10.5a	170.9 ± 23.4a	232.5 ± 14.5a	−60.4 ± 29.7a	69.1 ± 1.6a	110.6 ± 8.2a	−0.57 ± 0.32a	2.41 ± 0.24a	1.27 ± 0.14a
Ordinary *japonica* rice (C)	278.1 ± 35.0b	114.7 ± 14.7a	163.4 ± 27.5a	220.7 ± 23.4b	−57.4 ± 30.7a	69.2± 1.8a	105.6 ± 11.4b	−0.56 ± 0.33a	2.44 ± 0.25a	1.27 ± 0.15a
Ordinary *japonica* rice (O)	294.5 ± 28.5a	122.6 ± 11.1a	171.9 ± 24.5a	235.0 ± 15.8a	−59.5 ± 29.1a	69.0± 1.6a	112.4 ± 8.8a	−0.55 ± 0.31a	2.41 ± 0.23a	1.26 ± 0.14a
Low-amylose *japonica* rice (W)	313.4 ± 20.7a	98.7 ± 12.9a	214.7 ± 29.1a	184.0 ± 24.9a	−129.4 ± 41.4a	67.4 ± 0.7a	85.3 ± 12.6a	−1.59 ± 0.63a	3.22 ± 0.49a	1.33 ± 0.05a
Low-amylose *japonica* rice (C)	282.8 ± 19.7b	88.2 ± 10.8b	194.6 ± 29.5b	168.9 ± 21.9b	−113.9 ± 40.9b	66.8 ± 2.3a	80.7 ± 11.4b	−1.48 ± 0.63b	3.25 ± 0.54a	1.70 ± 0.30b
Low-amylose *japonica* rice (O)	305.6 ± 31.5a	96.3 ± 13.2a	209.2 ±34.6a	181.6 ± 25.1a	−124.0 ± 46.2a	66.9 ± 1.1a	85.2 ± 13.0a	−1.53 ± 0.67a	3.21 ± 0.51a	1.71 ± 0.30b

Pasting properties of each rice group, such as the premium *Japonica* rice Koshihikari (*n* = 15), ordinary *Japonica* rice (*n* = 35), and low-amylose *Japonica* rice (*n* = 4), are shown in the same lane. Different letters (a, b) mean that whole, chalky and original grains among each group samples are significantly different. Abbreviation: Max. vis, maximum viscosity; Mini. vis, minimum viscosity; Pt, pasting temperature; SB, setback (Final.vis − Max. vis); BD, breakdown (Max. vis − Mini. vis); Cons, consistency (Fin. vis − Mini. vis); Fin. vis, final viscosity; W, whole grains; C, chalky grains; and O, original grains. Values are shown as mean ± standard deviation.

**Table 2:** Analysis of iodine absorption parameters of whole and chalky rice grains in 54 *Japonica* rice in 2020.

	AAC (%)	λ_max_ (nm)	A_λmax_	λ_max_/AAC	λ_max_/A_λmax_	Fb_3_ (%)
Premium *japonica* rice (W)	15.6 ± 0.6a	557.4 ± 6.2a	0.280 ± 0.011a	35.9 ± 1.5a	1994.3 ± 68.8a	11.7 ± 0.5a
Premium *japonica* rice (C)	14.7 ± 1.3a	564.9 ± 3.5a	0.257 ± 0.012b	38.7± 3.5a	2202.5 ± 101.1b	10.7 ± 0.5a
Ordinary *japonica* rice (W)	15.3 ± 1.6a	558.4 ± 7.7a	0.282 ± 0.017a	37.0 ± 3.8a	1985.8 ± 111.0a	11.8 ± 0.8a
Ordinary *japonica* rice (C)	14.7 ± 1.9a	555.9 ± 8.7a	0.276 ± 0.020a	38.5 ± 4.5a	2022.2 ± 132.1a	11.6 ± 0.9a
Low-amylose rice (W)	12.1 ± 1.3a	542.4 ± 6.9a	0.248 ± 0.012a	45.3 ± 4.1a	2194.2 ± 79.1a	10.3 ± 0.5a
Low-amylose rice (C)	10.7 ± 1.2b	533.6 ± 6.4b	0.230 ± 0.008b	51.3 ± 5.8a	2325.8 ± 57.7a	9.5 ± 0.4a

AAC, λ_max_, A_λmax_, and Fb_3_ were measured, and the ratios of A_λmax_ to AAC or λ_max_ to A_λmax_ were calculated. In Table 2, data of premium *Japonica* rice Koshihikari (*n* = 15), ordinary *Japonica* rice (n = 35), and low-amylose *Japonica* rice (*n* = 4) are shown in the same lanes. Additionally, difference between whole grains and chalky grains in the same column were compared. Different letters (a, b) mean that whole and chalky grains in each same rice samples are significantly different. Abbreviation: AAC, apparent amylose content; λmax, peak wavelength on iodine staining; Aλmax, absorbance at λmax; Fb_3_, proportion of long chains in amylopectin (DP ≥ 37)%; Values are shown as mean ± standard deviation.

**Table 3:** Correlation between the ratios of *α*-amylase activities of chalky unpolished grains to those of whole ones and pasting properties in original 50 *Japonica* rice except low-amylose *Japonica* rice.

	Max. vis		Mini. vis		BD		Fin. vis		SB		Pt		Cons		Set/Cons		Max/Mini		Max/Fin	Ratios
																				α-Amylase (C/W)
Max. vis	1.00	-																		
Mini. vis	0.47	**	1.00	-																
BD	0.93	**	0.11		1.00	-														
Fin. vis	0.16		0.85	**	−0.18		1.00	-												
SB	−0.86	**	−0.01		−0.97	**	0.36	**	1.00	-										
Pt	0.48	**	0.14		0.47	**	−0.02		−0.46	**	1.00	-								
Cons	−0.29	*	0.29	*	−0.45	**	0.75	**	0.66	**	−0.21		1.00	-						
Set/Cons	−0.81	**	0.05		−0.93	**	0.44	**	0.99	**	−0.43	**	0.73	**	1.00	-				
Max/Mini	0.60	**	−0.42	**	0.85	**	−0.60	**	−0.87	**	0.35	*	−0.56	**	−0.87	**	1.00	-		
Max/Fin	0.79	**	−0.11		0.94	**	−0.47	**	−0.99	**	0.42	**	−0.71	**	−1.00	**	0.91	**	1.00	
ratios of α-amylase (C/W)	−0.11		−0.51	**	0.09		−0.60	**	−0.20		0.15		−0.45	**	−0.25		0.35	*	0.27	1.00

* Correlation is significant at 5% (*) by *t*-test. ** Correlation is significant at 1% by *t* test.

## Data Availability

The datasets generated for this study are available on request to the corresponding author.

## Supplementary Materials

The following supporting information can be downloaded at https://www.mdpi.com/article/10.3390/foods11213422/s1, Figure S1: Comparison between ratio of omega-6/omega-3 (n-6/n-3) of chalky and whole rice grains of 54 *Japonica* brown rice samples in 2020. Table S1: Pasting properties of whole grains in 54 *Japonica* brown rice in 2020. Table S2: Pasting properties of chalky grains in 54 *Japonica* brown rice in 2020. Table S3: Pasting properties of original brown rice samples in 54 *Japonica* brown rice in 2020. Table S4: Analysis of iodine absorption parameters of whole rice grains in 54 *Japonica* rice in 2020. Table S5: Analysis of iodine absorption parameters of chalky rice grains in 54 *Japonica* rice in 2020. Table S6: Physical properties of boiled rice of 100% whole rice grains in 19 kinds of *Japonica* rice in 2020. Table S7: Physical properties of boiled rice of 30% chalky rice blended rice grains in 19 kinds of *Japonica* rice in 2020. Table S8: Correlation between whole and chalky rice grains with the results of physical parameters of boiled rice, iodine analysis, pasting properties, α-amylase activities, sugar contents, l-glutamic acid, and l-amino acid of 19 kinds of *Japonica* rice in 2020. Table S9: Sugar contents of boiled rice with 100% whole rice grains and 30% chalky rice blended grains in 19 kinds of *Japonica* rice in 2020.

## Abbreviations

AAC; apparent amylose content; λ_max_; peak wavelength on iodine staining; Aλ_max_; absorbance at λ_max_; Fb_3_, proportions of long chains in amylopectin (DP ≥ 37%); CD, chain length distribution; RS, resistant starch; SLC, super-long chains; RVA, rapid visco analyser; SB, setback; BD, breakdown; Max. vis., maximum viscosity; Mini. vis., minimum viscosity; Pt, pasting temperature; Cons, consistency; Fin. vis., final viscosity; SB/Cons, setback/consistency; Max/Min, maximum viscosity/minimum viscosity; Max/Fin, maximum viscosity/final viscosity; Whole rice, 100% whole rice grains; Blend rice, 30% chalky grain blended rice.

## References

[1] Morita S., Shiratsuch I.H., Takahashi J., Fujita K. (2004). Effect of temperature on grain ripening in rice plants. Jpn. Crop Sci..

[2] Nakata M., Fukamatsu Y., Miyashita T., Hakata M., Kimura R., Nakata Y., Kuroda M., Yamaguchi T., Yamakawa H. (2017). High temperature-induced expression of rice α-amylase in developing endosperm produces chalky grains. Front. Plant Sci..

[3] Mitsui T., Shiraya S., Kaneko K., Wada K. (2016). Proteomics of rice grain under high temperature stress. Plant Prod. Sci..

[4] Liao J.-L., Zhou H.-W., Zhang H.-Y., Zhong P.-A., Huang Y.-J. (2014). Comparative proteomic analysis of differentially expressed proteins in the early milky stage of rice grains during high temperature stress. J. Exp. Bot..

[5] Nevam A.Y.M., Emon R.M., Malek M.A., Hasan M.M., Alam M.A., Muharam M., Aslani F., Rafii M.Y., Ismail M.R. (2018). Relationship between high temperature and formation of chalkiness and their effects on quality of rice. Biomed. Res. Int..

[6] Mitsui T., Yamakawa H., Kobata T. (2016). Molecular physiological aspects of chalking mechanism in rice grains under high-temperature stress. Plant Prod. Sci..

[7] Asaoka M., Okuno K., Sugimoto Y., Kawakami J., Fuwa H. (1984). Effect of environmental temperature during development of rice plants on some properties of endosperm starch. Starch-Stärke.

[8] Ahmed N., Tetlow I., Nawaz S., Iqbal A., Mubin M., Rehman M.S.N., Butt A., Lighfoot D.A., Maekawa M. (2014). Effect of high temperature on grain filling period, yield, amylose content and activity of starch biosynthesis enzymes in endosperm of basmatic rice. J. Sci. Food Agric..

[9] Bergman C.J., Bhattacharya K.R., Ohtsubo K., Champagne E.T. (2004). Rice end-use quality analysis. Rice-Chemistry and Technology.

[10] Blakeney A.B., Welsh L.A., Bannon D.R., Martin D.J., Wrigley C.W. (1991). Rice quality analysis using a computer controlled RVA. Cereals International.

[11] Champagne E.T., Bett K.L., Vinyard B.T., McClung A.M., Barton F.E., Moldenhauer K., Linscombe S., Mckenzie K. (1999). Correlation between cooked rice texture and rapid visco analyzer measurements. Cereal Chem..

[12] Ohtsubo K., Nakamura S., Li J. (2017). Evaluation of palatability of cooked rice. Adovances in International Rice Research.

[13] Nakamura S., Satoh A., Aizawa M., Ohtsubo K. (2022). Characteristics of physicochemical properties of chalky grains of *Japonica* rice generated by high temperature during ripening. Foods.

[14] Cereals and Grains Association (2022). AACC Approved Methods of Analysis, 11th Ed., 44-15.02 Moisture—Air-Oven Methods. https://www.cerealsgrains.org/resources/Methods/Pages/44Moisture.aspx.

[15] Yamamoto K., Sawada S., Onogaki I. (1981). Effects of quality and quantity of alkali solution on the properties of rice starch. Denpun Kagaku.

[16] Toyoshima H., Okadome H., Ohtsubo K., Suto M., Horisue N., Inatsu O., Narizuka A., Aizaki M., Inouchi N., Fuwa H. (1997). Cooperative test on the small-scale rapid method for the gelatinization properties test of rice flours with a rapid visco analyser (in Japanese). Nippon. Shokuhin Kogakukaishi.

[17] Nakamura S., Katsura J., Kato K., Ohtsubo K. (2016). Development of formulae for estimating amylose content and resistant starch content based on the pasting properties measured by RVA of *Japonica* polished rice and starch. Biosci. Biotechnol. Biochem..

[18] Juliano B.O. (1971). A simplified assay for milled-rice amylose. Cereal Sci. Today.

[19] Nakamura S., Satoh H., Ohtsubo K. (2015). Development of formulae for estimating amylose content, amylopectin chain length distribution, and resistant starch content based on the iodine absorption curve of rice starch. Biosci. Biotechnol. Biochem..

[20] Nakamura S., Yamaguchi H., Benitani Y., Ohtsubo K. (2020). Development of a novel formula for estimating the amylose content of starch using *Japonica* milled rice flours based on the iodine absorption curve. Biosci. Biotechnol. Biochem..

[21] Odahara M., Sokooshi H., Takahashi T., Okadome H., Ohtsubo K. (2004). The effect of sushi vinegar on texture of sushi rice before and after storage under low temperature. Nippon Shokuhin Kagaku Kogaku Kaishi.

[22] Juliano B.O., Juliano B.O. (1985). Criteria and tests for rice grain qualities. Rice-Chemistry and Technology.

[23] Hizukuri S., Takeda Y., Maruta N., Juliano B.O. (1989). Molecular structure of rice starch. Carbohydr. Res..

[24] Li H., Yu L., Yu W., Li H., Gilbert R. (2019). Autoclaved rice: The textural property and its relation to starch leaching and the molecular structure of leached starch. Food Chem..

[25] Nakamura S., Katsura J., Maruyama Y., Ohtsubo K. (2019). Relationship between fatty acid composition and starch properties of 30 *japonica* rice cultivars. Cereal Chem..

[26] Nakamura S., Katsura J., Maruyama Y., Ohtsubo K. Relationship between fatty acid composition, pasting properties and iodine analysis of *japonica* rice cultivars. Proceedings of the 2018 Annual Meeting of Japan Society for Bioscience Biotechnology, and Agrochemistry.

[27] Simopoulos A.P. (2002). The important of the ratio of omega-6/omega-3 essential fatty acids. Biomed. Pharmacother..

[28] Taira H., Nakagahra M., Nagamine T. (1988). Fatty acid composition of Indica, Sinica, Javanica, and *Japonica* groups of nonglutinous brown rice. J. Agric. Food Chem..

[29] Raphaelides S., Karkalas J. (1988). Thermal dissociation of amylose-fatty acid complexes. Carbohydr. Res..

[30] Goderis B., Putseys J.A., Gommes C.J. (2014). The structure and thermal stability of amylose-lipid complexes. A Case Study Amylose-Glycerol. Monostearate Cryst. Growth Des..

[31] Umemoto T., Terashima K., Nakamura Y., Satoh H. (1999). Differences in amylopectin structure between two rice varieties in relation to the effects of temperature during grain-filling. Starch-Stärke.

[32] Asaoka M., Okuno K. (1985). Effect of environmental temperature at the milky stage on amylose content and fine structure of amylopectin of waxy and nonwaxy endosperm starches of rice. Agric. Biol. Chem..

[33] Takami K., Koriyama T., Ohtsubo K. (1998). Staling characteristics of cooked low-amylose rice and a proposal of evaluation method. Nippon Shokuhin Kagaku Kogaku Kaishi.

[34] Umemoto T., Horibata T., Aoki N., Hiratsuka M., Yano M., Inouchi N. (2008). Effects of variations in starch synthase on starch properties and eating quality of rice. Plant Prod. Sci..

[35] Gallant D.J., Bouchet B., Baldwin P.M. (1997). Microscopy of starch evidence of a new level of granule organization. Carbohydr. Polym..

[36] Inouchi N., Ando H., Asaoka M., Okuno K., Fuwa H. (2000). The effect of environmental temperature on distribution of unit chains of rice amylopectin. Starch/Strake.

[37] Igarashi T., Yanagihara T., Kanda H., Kawamoto K., Masaki K. (2009). Development of new eating quality evaluation method based on iodine adsorption multispectral analysis of rice flour. J. Crop Sci..

[38] Yano M., Okuno K., Satoh H., Omura T. (1988). Chromosomal location of genes conditioning low amylose content of endosperm starches in rice, *Oryza sativa* L.. Theor. Appl. Genet..

[39] Tateyama M., Sakai M., Suto M. (2005). Varietal differences in the response of the amylose content of the endosperm of low-amylose rice (*Oryza sativa* L.) Lines to temperature during the ripening period. Breed. Res..

[40] Sato H., Suzuki Y., Sakai M., Imbe T. (2002). Molecular characterization of *Wx-mq*, a novel mutant gene for low-amylose content in endosperm of rice (*Oryza sativa* L.). Breed. Res..

[41] Yamakawa H., Hakata M. (2010). Atlas of rice grain filling-related metabolism under high temperature joint analysis of metabolome and transcriptome demonstrated inhibition of starch accumulation and induction of amino acid accumulation. Plant Cell Physiol..

[42] Yamakawa H., Hirai-Kimura R., Nakata Y., Nakata M., Kuroda M., Yamaguchi T. (2017). An activity-staining method on filtration paper enables high-throughput screening of temperature-sensitive and inactive mutations of rice α-amylase for improvement of rice grain quality. Plant Cell Physiol..

[43] Patindol J., Wang Y.-J. (2003). Fine structures and physicochemical properties of starches from chalky and translucent kernels. J. Agric. Food Chem..

[44] IPCC 6th Assessment Report. https://www.ipcc.ch/report/ar6/wg1/downloads/report/IPCC_AR6_WGI_SPM_final.pdf.

[45] Hakata M., Kuroda M., Miyashita T., Yamaguchi T., Kojima M., Sakakibara H., Mitsui T., Yamakawa H. (2012). Suppression of α- amylase genes improve quality of rice grain ripened under high temperature. Plant Biotechnol. J..

[46] Iwata H., Iwase S., Takahama K., Matuura H., Itani T., Aramaki I. (2001). Relation between α-glucosidase activity and physical and chemical properties of rice. Nippon Shokuhin Kagaku Kogaku Kaishi.

[47] Nakamura S., Katsura J., Maruyama Y., Ohtsubo K. (2021). Evaluation of hardness and retrogradation of cooked rice based on its pasting properties using a novel RVA testing. Foods.

[48] Okadome H., Toyoshima H., Ohtsubo K. (1999). Multiple measurements of physical properties of individual cooked rice grains with a single apparatus. Cereal Chem..

[49] Awazuhara M., Nakagawa A., Yamaguch J., Fujiwara T., Hayashi H., Hatae K., Chino M., Shimada A. (2000). Distribution and characterization of enzymes responsible for starch degradation in rice. (Oryza sativa Cv. Koshihikari). J. Agric. Food Chem..

[50] Kasai M., Ishiguro K., Kyoda H., Hamazono T., Hatae K., Shimada A. (2000). Change in the amounts of reducing sugars and free amino acids in rice during the cooking processes. JSHE.

[51] Abe K., Kondo H., Arai S. (1987). Purification and properties of a cysteine proteinase from germinating rice seeds. Agric. Biol. Chem..

[52] Doi E., Komori N., Matoba T., Morita Y. (1980). Some properties of carboxypeptidases in germinating rice seeds and rice leaves. Agric. Biol. Chem..

[53] Doi E., Komori N., Matoba T., Morita Y. (1980). Carboxypeptidase like metal enzyme in rice seedlings. Agric. Biol. Chem..

[54] Tashiro M., Kurata A., Maki Z. (1990). Occurrence of cysteine proteinase inhibitors in cereals. Sci. Rep. Kyoto Prefect. Univ..

[55] Tamura S., Kenmochi K. (1963). Studies on amino acid content of rice (part III). Biosci. Biotechnol. Biochem..

[56] Matsuzaki A., Takano T., Sakamoto S., Kuboyama T. (1992). Relation between eating quality and chemical components in milled rice and amino acid contents in cooked rice. J. Crop Sci..

[57] Masumura T., Shigemitu T., Goto F., Saito Y., Ishimaru T., Kondo M. (2013). The influence that a high temperature stress gives to the structure of seed storage protein and the taste of cooked rice. J. Crop Sci..

[58] Yamakawa H., Hirose T., Kuroda M., Yamaguchi T. (2007). Comprehensive expression profiling of rice grain filling-related genes under high temperature using DNA microarray. Plant Physiol..

[59] Juliano B.O., Juliano B.O. (1985). Biochemical properties of rice. Rice-Chemistry and Technology.

[60] Ikeda A., Ueguchi-Tanaka M., Sonoda H., Kitano M., Koshioka Y., Futsuhara M., Matsuoka M., Yamaguchi I. (2001). slender rice, a constitutive gibberellin response mutant, is caused by a null mutation of the SLR_1_, an ortholog of the height-regulating gene GAI/RGA/D8. Plant Cell.

[61] Ueguchi-Tanaka M., Ashikari M., Nakajima M., Itoh H., Katoh E., Kobayashi M., Chow T.Y., Hsing Y.C., Kitano H., Yamaguchi I. (2005). *GIBBERELLIN INSENSITIVE DWARF1* encodes a soluble receptor for gibberellin. Nature.

