# Supplementary material for: Palatability and Bio-Functionality of Chalky Grains Generated by High-Temperature Ripening and Development of Formulae for Estimating the Degree of Damage Using a Rapid Visco Analyzer of Japonica Unpolished Rice
Source: Foods. 2022 Oct 28;11(21):3422. doi: 10.3390/foods11213422 (PMC9658192; doi:10.3390/foods11213422)
Supplement: Supplementary file 1 [file foods-11-03422-s001.zip › figure S1.pptx]

## Slide 1
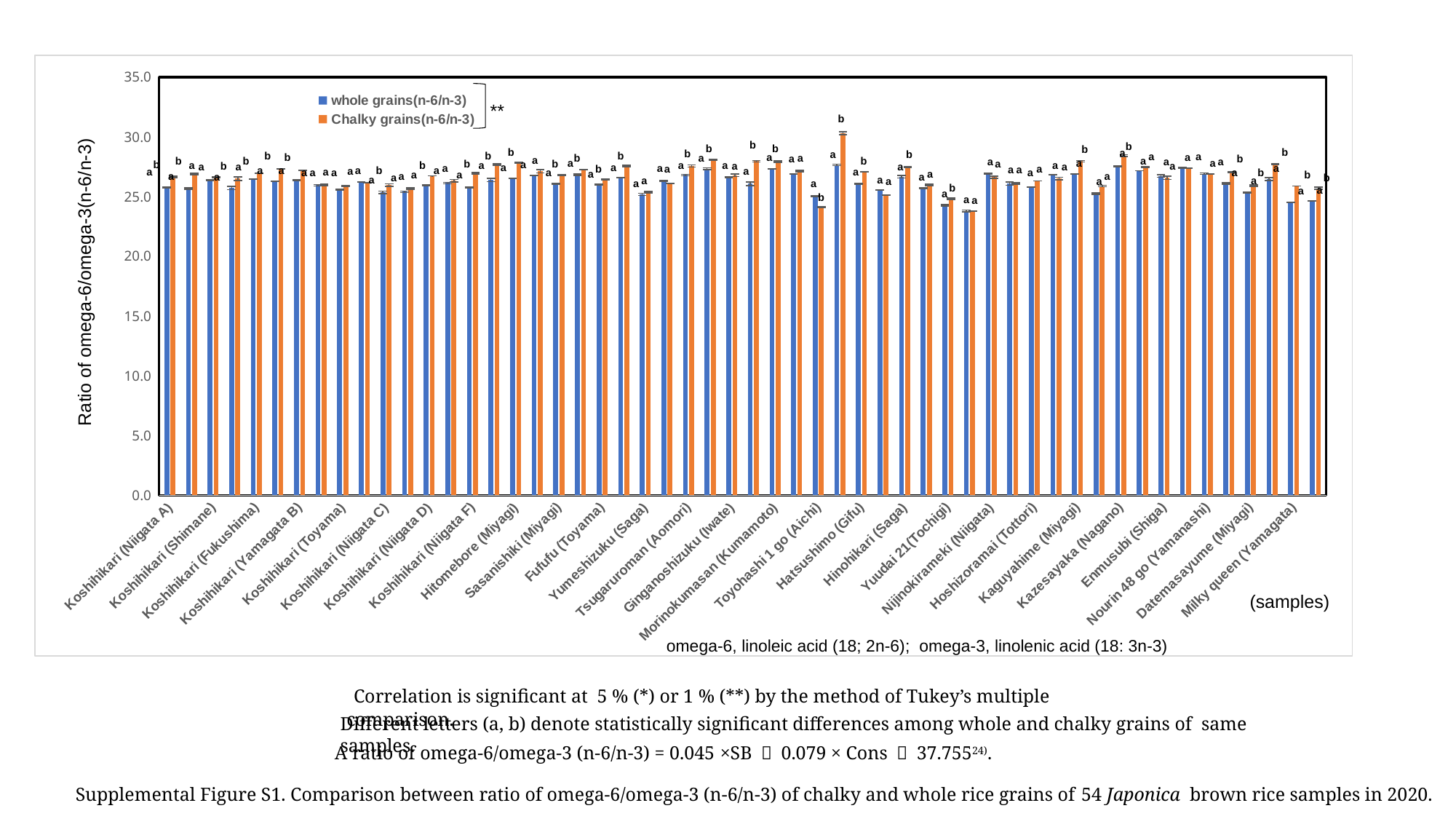

### Chart
| Category | whole grains(n-6/n-3) | Chalky grains(n-6/n-3) |
|---|---|---|
| Koshihikari (Niigata A) | 25.749252573056147 | 26.652943701757266 |
| Koshihikari (Niigata B) | 25.665149760163604 | 26.89197161997238 |
| Koshihikari (Shimane) | 26.385610967523863 | 26.59266368187347 |
| Koshihikari (Saga) | 25.716884142161945 | 26.492415303501637 |
| Koshihikari (Fukushima) | 26.468619520801816 | 26.937280137437607 |
| Koshihikari (Yamagata A) | 26.298495860355615 | 27.33788833994029 |
| Koshihikari (Yamagata B) | 26.36573833562144 | 27.190908721280277 |
| Koshihikari (Ibaragi) | 25.94714396582309 | 25.977479139408587 |
| Koshihikari (Toyama) | 25.598896668835998 | 25.890343374589182 |
| Koshihikari (Kyoto) | 26.21790114642552 | 26.155764992251143 |
| Koshihikari (Niigata C) | 25.3538131574471 | 25.960836602087607 |
| Koshihikari (Yamanashi) | 25.402977162147558 | 25.667297000382092 |
| Koshihikari (Niigata D) | 25.954739200762734 | 26.72953380144032 |
| Koshihikari (Niigata E) | 26.132847966878575 | 26.313024278641425 |
| Koshihikari (Niigata F) | 25.755165969564842 | 26.955284026351283 |
| Shinnosuke (Niigata) | 26.391399961568418 | 27.673701665723026 |
| Hitomebore (Miyagi) | 26.514041528328104 | 27.838884157412505 |
| Oidemai (Kagawa) | 26.79121606795347 | 27.129484019246117 |
| Sasanishiki (Miyagi) | 26.060199450208707 | 26.806847396530127 |
| Kinumusume (Shimane) | 26.828267153719583 | 27.240836862383546 |
| Fufufu (Toyama) | 26.022466640895374 | 26.43959693627694 |
| Akitakomachi (Akita) | 26.580417289533145 | 27.544446945898226 |
| Yumeshizuku (Saga) | 25.183429250529407 | 25.36143762813105 |
| Tsuyahime (Shimane) | 26.29810380048174 | 26.108065627399075 |
| Tsugaruroman (Aomori) | 26.79835561006211 | 27.55427035536583 |
| Sagabiyori (Saga) | 27.32912840321388 | 28.087221958790323 |
| Ginganoshizuku (Iwate) | 26.611598592111285 | 26.78390102653873 |
| Ichihomare (Fukui) | 26.066370193447245 | 27.956047290260322 |
| Morinokumasan (Kumamoto) | 27.322649672960722 | 27.929260400076206 |
| Seitennohekireki (Aomori) | 26.886080686284537 | 27.124553124877725 |
| Toyohashi 1 go (Aichi) | 25.023085951661837 | 24.097367923875694 |
| Sasashigure (Miyagi) | 27.642583612127765 | 30.298791032412833 |
| Hatsushimo (Gifu) | 26.071611227682624 | 27.052954689070063 |
| Tennotsubu (Fukushima) | 25.540796097239692 | 25.1126472151214 |
| Hinohikari (Saga) | 26.649074474960415 | 27.48055896767795 |
| Haenuki (Yamagata) | 25.719175949647045 | 25.9700805905664 |
| Yuudai 21(Tochigi) | 24.268389266713676 | 24.819139002012562 |
| Harumi (Kanagawa) | 23.798141412934992 | 23.77711481019129 |
| Nijinokirameki (Niigata) | 26.89140590225367 | 26.618604022799268 |
| Hitomebore (Miyagi) | 26.07828646257569 | 26.088645295737273 |
| Hoshizoramai (Tottori) | 25.774933762082398 | 26.31082654330419 |
| Tsukiakari (Niigata) | 26.844653475174155 | 26.51245202945838 |
| Kaguyahime (Miyagi) | 26.88211964906668 | 27.955397318224872 |
| Akisakari (Fukui) | 25.235907111279364 | 25.87684561925243 |
| Kazesayaka (Nagano) | 27.543302227456383 | 28.425654388111454 |
| Satojiman (Kanagawa) | 27.154299588783477 | 27.4651523574966 |
| Enmusubi (Shiga) | 26.73016326099067 | 26.563113266050387 |
| Nipponbare (Shiga) | 27.42405658191906 | 27.36801846708431 |
| Nourin 48 go (Yamanashi) | 26.918314404034824 | 26.897834137388166 |
| Tsuyahime (Yamagata) | 26.104598169432656 | 27.051852307919443 |
| Datemasayume (Miyagi) | 25.355752270024947 | 25.90730452099579 |
| Yumepirika (Hokkaido) | 26.461851982639054 | 27.702571213762692 |
| Milky queen (Yamagata) | 24.49344700084039 | 25.88795975668306 |
| Milky queen (Kyoto) | 24.646991191490972 | 25.69457739723465 |
**
b
b
b
b
b
b
b
b
a
b
a
b
b
b
b
a
a
a
a
b
a
a
b
b
a
a
a
b
b
b
a
a
a
a
a
a
b
b
a
b
a
b
a
a
a
a
a
a
a
b
a
a
a
a
a
a
a
a
a
b
a
a
a
a
a
a
a
b
a
a
a
a
a
a
a
b
a
a
a
a
a
a
a
a
a
b
a
a
a
a
a
b
a
a
a
a
a
a
a
a
a
b
a
a
a
b
a
a
Ratio of omega-6/omega-3(n-6/n-3)
(samples)
 omega-6, linoleic acid (18; 2n-6); omega-3, linolenic acid (18: 3n-3)
 Correlation is significant at 5 % (*) or 1 % (**) by the method of Tukey’s multiple comparison.
Different letters (a, b) denote statistically significant differences among whole and chalky grains of same samples.
A ratio of omega-6/omega-3 (n-6/n-3) = 0.045 ×SB － 0.079 × Cons ＋ 37.75524).
Supplemental Figure S1. Comparison between ratio of omega-6/omega-3 (n-6/n-3) of chalky and whole rice grains of 54 Japonica brown rice samples in 2020.
